# Supplementary material for: Pediatric diarrhea patients living in urban areas have a higher incidence of Clostridioides difficile infection
Source: PLOS Glob Public Health. 2023 Jan 24;3(1):e0000477. doi: 10.1371/journal.pgph.0000477 (PMC10022077; doi:10.1371/journal.pgph.0000477)
Supplement: Attachment — Submitted filename: Response to Reviewers comments _09.23.22.docx [file pgph.0000477.s001.docx]

**Manuscript Title:**	**Pediatric Diarrhea Patients Living in Urban Areas Have Higher Incidence of *Clostridioides difficile* Infection**

**Manuscript ID:	PGPH-D-22-00619**

**Response to Reviewers’ Comments**

|  | **Reviewers’ comments** | **Authors’ responses** |
| --- | --- | --- |
|  | **Reviewer One** |  |
| 1. | In the abstract please mention your study design, and a sentence about the statistical methods you used. | A statement has been added in the abstract describing the applicable study design and the statistical methods used for data analysis. |
| 2. | Study Population: Please include your study design. | The study design used has been included in the abstract and in the methods section. |
| 3. | How did you determine this sample size? Please describe your sampling procedure. And justify whether 76 sample size provide the adequate level of power for this study. | The estimated sample size was determined using the Design of Experiment (DOE) module of the SAS JMP Statistical Discovery^TM^ Software. The sample size of n=76 was assessed as providing an adequate level of statistical power being > 95%. A detailed description of the sampling procedures adopted at the participating hospitals is outlined in the methods section under the “*Study population and sample collection*” subsection of the methods section. |
| 4. | Since you have considered three different pediatric groups as your study participant, so how did you adjust for their individual effects on CDI? | We adjusted for independent factors of interest and their associations using the partition-based model based on the participant’s area of residence, age category, and sex to determine the probability of obtaining a positive CDI test result in the study population (see Table 3). |
| 5. | It is important to know whether your sample size is adequate enough for performing the statistical test and showing sufficient statistical power to answer your research objectives. | The authors agree with the reviewer’s comments on the importance of adequacy of sample size in relation to statistical power and test. However, as part of the experimental design and based on our research objectives, we initially determined the sample size. The sample size of n=76 was considered adequate and established to have a statistical power of 98.91% at a probability level of 0.05 to detect a difference in CDI test outcome.   |
| 6. | In line 151, you have mentioned that the frequency distribution of study sample is shown by race/ ethnicity which didn't display in the results section. | We thank the reviewer for an insightful observation. Race/ethnicity was not considered an independent factor in the current study because the majority of the sample was of the same race. The statement on line 151 related to race/ethnicity has been deleted accordingly. |
| 7. | The level of statistical significance was considered as <=0.05 in this study. According to this cut off-point, the association between knowledge of antibiotics and CDI occurrence are not statistically significant at 5% level of significance. | The authors appreciate the point raised by the reviewer. However, it was assumed to be significant based on the standardized form since the third decimal point is below a convertible point. We have decided to use the standardized significance levels throughout the manuscript in line with what was indicated in the methods section. |
| 8. | In line 197-200, please describe a little more about the patterns exhibits in the figure 2a, 2b. | The patterns displayed on figures 2a and 2b have been further described in the results section to enhance better understanding and improve clarity. |
| 9. | In figure 2, please specify the color legends. | Per the reviewer’s comment, the color legends for Figure 2 have been revised. |
| 10. | Please work on the grammar and sentence structure of the manuscript | We have reviewed the manuscript for both grammatical and sentence structure issues. Changes including rephrasing and/or corrections were made, where necessary. |
|  | **Reviewer Two** |  |
| 1. | The authors should highlight the reasons for the non-diagnosis of CDI in Nigeria from the health system perspective also. Is it a shortage of trained staff or sophisticated equipment which are expensive or so on? This should be included in the discussion section. | We already included the points highlighted by the reviewer in the introduction section of the paper. As requested, we have also revised the discussion section addressing the points raised. |
| 2 | Secondly, as multiple diagnostic tests are mentioned in the paper, are there any standard treatment guidelines (STGs) for the diagnosis of CDI in Nigeria or other LMICs? if yes, details of diagnostics required for the diagnosis of CDI in a real-world scenario should be added in the introduction of the article. | There is STG by Nigeria’s federal ministry of health but highly unpopular among Nigeria clinicians according to a report by Ayinbuomwan and Isah. (2019). As a result, most of the clinicians in Nigeria rely on WHO STG or other international STG for diagnosing and treatment of their CDI patients. |
| 3. | Thirdly, as mentioned in the limitations why the presence of other diarrhoea-causing pathogens was not tested in this study? Does it mean that it can be a non-CDI infection but due to lack of testing classified as a CDI? Can be a multiple pathogen infection? If yes, how authors decided that it's a CDI and no other pathogen is predominant? | It is possible that other bacteria may be the cause of the diarrhoea or may co-habit with CDI, which we have reported in one of our previous studies (Adesoji and Liadi. 2020). However, this study focused mainly on CDI. A comprehensive study on CDI co-infections with other pathogens, antibiogram, and other risk factors will be carried out in future studies. That is why we mentioned under the study limitation that the presence of other diarrhea-causing pathogens was not tested in this study and will be the subject of our ongoing research in this population. |
| 4. | Lastly, the limitations section is written very briefly, more details can be added to it. | Per the reviewer’s suggestion, we have added more detail to the study limitation section of the paper. |
|  | **Academic Editor** | **Authors’ responses** |
| 1. | A rebuttal letter that responds to each point raised by the editor and reviewer(s). You should upload this letter as a separate file labeled 'Response to Reviewers'. | A rebuttal letter that responds to each point raised by the editor and reviewer(s) has been upload as a separate file and labeled *'Response to Reviewers'*. |
| 2. | A marked-up copy of your manuscript that highlights changes made to the original version. You should upload this as a separate file labeled 'Revised Manuscript with Track Changes'. | A marked-up copy of the manuscript that highlights changes made to the original version has been uploaded as a separate file labelled *'Revised Manuscript with Track Changes'* |
| 3. | An unmarked version of your revised paper without tracked changes. You should upload this as a separate file labeled 'Manuscript'. | An unmarked version of your revised paper without tracked changes has been upload as a separate file labelled '*Manuscript*'. |
|  | **Journal Requirements** |  |
| 1. | Please provide additional details regarding participant consent. In the ethics statement in the Methods and online submission information, please ensure that you have specified whether consent was written or verbal/oral. If consent was verbal/oral, please specify: 1) whether the ethics committee approved the verbal/oral consent procedure, 2) why written consent could not be obtained, and 3) how verbal/oral consent was recorded. If your study included minors, please state whether you obtained consent from parents or guardians in these cases. If the need for consent was waived by the ethics committee, please include this information | Per the journal requirements, additional information relating to the consent procedures have been provided and the ethics statement section has been updated accordingly. |
| 2. | Please send a completed 'Competing Interests' statement, including any COIs declared by your co-authors. If you have no competing interests to declare, please state "The authors have declared that no competing interests exist". Otherwise please declare all competing interests beginning with the statement "I have read the journal's policy and the authors of this manuscript have the following competing interests:" | The 'Competing Interests' statement has been inserted in appropriate section of the article and/or the journal form as applicable. |
| 3. | Please amend your detailed Financial Disclosure statement. This is published with the article. It must therefore be completed in full sentences and contain the exact wording you wish to be published.  a. State the initials, alongside each funding source, of each author to receive each grant. b. State what role the funders took in the study. If the funders had no role in your study, please state: “The funders had no role in study design, data collection and analysis, decision to publish, or preparation of the manuscript.” | The authors have addressed these requirements related to Financial Disclosure statement and those have been inserted in appropriate section of the article and/or journal form as applicable. |
| 4. | Please provide separate figure files in .tif or .eps format only and remove any figures embedded in your manuscript file. Please also ensure that all files are under our size limit of 10MB.  For more information about how to convert your figure files please see our guidelines: https://journals.plos.org/globalpublichealth/s/figures | All figures in the manuscript have been provided in separate files in the journal-approved format and size limit of 10 MB. The authors have also removed any figures previously embedded in the manuscript file. |
| 5. | Please provide a complete Data Availability Statement in the submission form, ensuring you include all necessary access information or a reason for why you are unable to make your data freely accessible. If your research concerns only data provided within your submission, please write "All data are in the manuscript and/or supporting information files" as your Data Availability Statement. | This journal request has been fulfilled and the required data availability statement has been provided in the appropriate section of this manuscript. The statement will also be included in the data availability section in the submission form. |

**References**

Adesoji, A.T. and Liadi, A.M., 2020. Antibiogram studies of *Escherichia coli* and *Salmonella* species isolated from diarrheal patients attending Malam Mande General Hospital Dutsin-Ma, Katsina State, Nigeria. The Pan African Medical Journal, 37.

Ayinbuomwan, A. S., & Isah, A. O. (2019). Standard treatment guidelines: Perception and utilization in a tertiary health care facility in South-South, Nigeria. *Annals of African Medicine*, *18*(1), 47.
